# Global Genomic Analysis of Bovine-Associated *Klebsiella pneumoniae* Reveals Genetic Diversity and Resistance–Virulence Profiles

**DOI:** 10.3390/biology15141215

**Published:** 2026-07-22

**Authors:** Meihui Tian, Yaqian Liang, Jia Lu, Weidi Shi, Yang Zhao, Weize Gan, Shuan Jia, Chencheng Xiao, Tianyi Zhao, Hui Zhang

**Affiliations:** 1 College of Animal Science and Technology, Shihezi University, Shihezi 832000, China; 2 Changji Prefecture Center for Animal Disease Prevention and Control, Changji 831100, China; 3 Xinjiang Uygur Autonomous Region Center for Animal Disease Prevention and Control, Urumqi 830011, China

**Keywords:** bovine-associated *Klebsiella pneumoniae*, microbial genomics, genetic diversity, antimicrobial resistance, virulence-associated genes, plasmid replicons, One Health

## Abstract

Bovine-associated *Klebsiella pneumoniae* is an important bacterial species linking animal health, microbial ecology, and One Health-oriented antimicrobial resistance research. In this study, we performed a global genomic analysis of 1291 publicly available bovine-associated *K. pneumoniae* genomes collected from 18 countries between 2005 and 2024 using data retrieved from NCBI. MLST, core-genome phylogenetic analysis, pangenome analysis, CARD, VFDB, and PlasmidFinder were used to characterize sequence types, genomic diversity, antimicrobial resistance-associated genes, virulence-associated genes, and plasmid replicons. A total of 256 sequence types were identified, among which ST107 was the most common. Core-genome phylogenetic analysis revealed multiple genomic lineages, while pangenome analysis identified 46,325 gene clusters, including 1967 core genes and 40,595 cloud genes, indicating an open pangenome structure and substantial accessory gene diversity. Virulence-associated genes were unevenly distributed, with *yagZ*/*ecpA* being the most frequently detected determinant. In total, 138 antimicrobial resistance-associated genes or potential resistance determinants were detected across 16 antimicrobial categories, including clinically important β-lactamase- and carbapenemase-associated genes. IncF-family plasmid replicons, particularly IncFIB(K)_1_Kpn3, were frequently detected, suggesting widespread plasmid replicon-associated genomic backgrounds; however, physical co-localization between resistance genes and specific plasmid backbones could not be confirmed. Overall, this study reveals the genetic diversity, resistance-associated gene reservoir potential, heterogeneity of virulence-associated genes, and plasmid replicon backgrounds of bovine-associated *K. pneumoniae*. Importantly, the genome-predicted AMR potential identified in this study should not be interpreted as confirmed phenotypic resistance without further experimental validation. These findings provide genomic insights for risk surveillance, candidate control-target screening, and microbiota-oriented intervention research.

## Simple Summary

*Klebsiella pneumoniae* is an important zoonotic opportunistic pathogen that can harbor antimicrobial resistance-associated genes and virulence-associated genes. Bovine-associated *K. pneumoniae* has attracted increasing attention because it may link cattle populations, farm environments, food production systems, and human health. In this study, we analyzed 1291 publicly available bovine-associated *K. pneumoniae* genomes collected from 18 countries between 2005 and 2024. The results showed that this bacterial population exhibited high genetic diversity and carried multiple categories of antimicrobial resistance-associated genes, virulence-associated genes, and plasmid replicon markers. Although some detected resistance-associated genes are clinically important, whether they confer actual phenotypic resistance requires further experimental validation. These findings suggest that bovine-associated *K. pneumoniae* may serve as a potential reservoir of antimicrobial resistance- and virulence-associated determinants, highlighting the need for continuous genomic surveillance within a One Health framework.

## 1. Introduction

*Klebsiella pneumoniae* is a representative opportunistic bacterial species within the genus *Klebsiella* of the family Enterobacteriaceae. It is characterized by broad host adaptability, substantial pathogenic potential, and a remarkable capacity to evolve antimicrobial resistance [1,2]. In livestock production, this bacterium has been associated with bovine pneumonia, mastitis, and septicemia, causing considerable economic losses to the global cattle industry. More importantly, key antimicrobial resistance genes carried by *K. pneumoniae*, including genes conferring resistance to β-lactams and carbapenems, may disseminate across livestock–environment–human interfaces, thereby making this species a potential reservoir of resistance determinants relevant to animal, environmental, and human health [3,4,5].

In recent years, the increasing availability of whole-genome sequencing has greatly advanced the molecular epidemiological investigation of *K. pneumoniae*. However, current genomic studies remain strongly human-centered, with more than 90% focusing on clinical genomes, whereas systematic investigations of bovine-associated strains are still limited [6]. Available but fragmented reports suggest that bovine-associated genomes exhibit marked geographic variation in antimicrobial resistance profiles and sequence types. Clinically important carbapenemase-associated genes, such as *bla*KPC and *bla*NDM, have been detected in genomes from major cattle-producing countries, including China and the United States. Nevertheless, global comparative analyses of genetic diversity, temporal genomic trends, and the co-occurrence of antimicrobial resistance and virulence-associated determinants remain insufficient [7,8,9]. Notably, plasmids represent an important genetic background for the dissemination of antimicrobial resistance genes in Enterobacteriaceae. However, common plasmid replicon types in bovine-associated *K. pneumoniae* and their co-detection patterns with antimicrobial resistance genes have not been systematically investigated. Because plasmid replicon detection alone cannot demonstrate physical co-localization between resistance genes and plasmid backbones, it is necessary to cautiously evaluate the potential association between plasmid replicon backgrounds and resistance determinants at the genomic level [10].

Therefore, in this study, we performed a comprehensive genome-wide analysis of bovine-associated *K. pneumoniae* genomes retrieved from the NCBI public database. By integrating molecular typing, antimicrobial resistance gene annotation, virulence-associated gene detection, and plasmid replicon analysis, we aimed to characterize the genetic diversity, virulence-associated gene profiles, resistance determinants, and plasmid replicon-associated genomic backgrounds of bovine-associated *K. pneumoniae*. We also sought to cautiously assess the potential co-occurrence between plasmid replicons and antimicrobial resistance genes. This study provides genomic evidence for understanding the biological characteristics of bovine-associated *K. pneumoniae* and evaluates its potential significance as a microbial reservoir of antimicrobial resistance and virulence-associated determinants within a One Health framework.

## 2. Materials and Methods

### 2.1. Genome Data Source, NCBI Search Strategy, and Screening of Bovine-Associated Genomes

All *K. pneumoniae* genome data used in this study were obtained from the public database of the National Center for Biotechnology Information (NCBI). To ensure transparency and reproducibility of the data retrieval process, the NCBI Assembly database was searched on 25 October 2025 using “*Klebsiella pneumoniae*” [Organism] as the initial search term. Publicly available *K. pneumoniae* genome assembly records, GenBank flat files in gbff format, and corresponding metadata were downloaded. The initial search retrieved 135,380 *K. pneumoniae*-associated genome records.

Subsequently, custom Python 3.9.12 scripts were used to parse and curate the downloaded gbff files and NCBI metadata tables. Key information was extracted for each genome, including assembly accession, assembly name, genome file name, NCBI genomic FNA download link, strain name, collection year, country or region of origin, host, isolation source, sample description, and downstream genomic feature profiles. These data were mainly derived from NCBI Assembly metadata tables, BioSample records, GenBank flat files, and the downstream MLST, antimicrobial resistance gene, virulence-associated gene, and plasmid replicon analyses performed in this study.

Bovine-associated genomes were identified using a three-step strategy consisting of source-term keyword screening, metadata cross-checking, and manual curation. First, bovine-associated terms were searched across the host, isolation source, source, sample title, and BioSample description fields. The inclusion keywords included bovine, cattle, cow, calf, dairy cattle, dairy cow, *Bos taurus*, bovine milk, cow milk, dairy milk, and bovine mastitis. Second, records matching these keywords were cross-checked to confirm that the host or sample source was associated with cattle, dairy cattle, calves, bovine milk, or bovine mastitis. Records described only by broad terms such as milk, mastitis, animal, or livestock were not included in the final dataset unless their bovine origin could be further confirmed using the host, isolation source, sample title, or BioSample description fields. For records with inconsistent metadata across different fields, the host and isolation source fields in BioSample were used as the primary criteria, together with sample title, sample description, and the GenBank source feature for manual verification.

Duplicate records were removed mainly based on assembly accession, BioSample accession, assembly name, and strain name. First, identical assembly accessions were retained only once. Second, when multiple assembly records corresponded to the same BioSample accession, they were regarded as duplicate assembly versions of the same isolate, and only one representative genome was retained. Representative assemblies were selected according to the following priority order: RefSeq assemblies were prioritized over corresponding GenBank assemblies, followed by higher assembly level, fewer contigs, higher N50, genome size within or close to the common genome-size range of *K. pneumoniae*, and more recent database update date. The typical reference genome-size range was approximately 4.5–6.5 Mb. For records with the same strain name but different BioSample accessions, collection year, country or region, host, isolation source, and assembly accession were manually checked. If they could not be confidently identified as duplicate records from the same isolate, they were retained as independent genomes. After source-term screening, metadata verification, and duplicate removal, 1291 bovine-associated *K. pneumoniae* genomes were retained for downstream analyses.

To ensure data traceability, a complete accession and metadata correspondence table was established. The complete accession numbers, assembly names, genome file names, NCBI download links, source metadata, MLST results, antimicrobial resistance gene profiles, virulence-associated gene profiles, and plasmid replicon profiles of the 1291 bovine-associated *K. pneumoniae* genomes included in the final dataset are provided in Table S1. This study was a retrospective bioinformatics analysis based entirely on publicly available genome assembly data; no additional bacterial genomes were collected or sequenced. All downstream genomic analyses were performed using publicly available genome assembly sequences and their associated annotation and metadata files, and raw sequencing reads were not used. Specifically, genome assembly sequence files (genomic FASTA, .fna) were used for MLST, antimicrobial resistance gene detection, virulence-associated gene detection, plasmid replicon detection, and species confirmation. For pangenome and core-genome phylogenetic analyses, all genome assemblies were uniformly annotated using Prokka v1.14.6 with default parameters to generate standardized GFF3 annotation files, which were then used as input for Roary. GenBank flat files (.gbff) were mainly used for genome annotation extraction, genome file verification, and metadata cross-checking. NCBI Assembly metadata and BioSample metadata were used to curate collection year, country or region, host, isolation source, sample description, and assembly-quality information.

To improve reproducibility of the metadata-based screening process, the custom Python workflow was further summarized as follows. First, NCBI Assembly metadata, BioSample metadata, and GenBank source fields were parsed and merged according to assembly accession and BioSample accession. Second, bovine-associated candidate records were identified using predefined keywords in the host, isolation source, sample title, BioSample description, and GenBank source fields. Third, records containing only broad or ambiguous source terms, such as “milk”, “mastitis”, “animal”, or “livestock”, were manually reviewed and retained only when bovine origin could be confirmed by at least one additional metadata field. Fourth, duplicate records were removed using assembly accession, BioSample accession, assembly name, and strain name. Finally, records with conflicting or incomplete metadata were manually curated, with the BioSample host and isolation source fields used as the primary criteria. This workflow was used to generate the final dataset of 1291 bovine-associated *K. pneumoniae* genomes.

### 2.2. Genome Quality Control and Species Confirmation

To ensure the reliability of the genome dataset included in this study, all candidate genomes were subjected to assembly-quality assessment and species confirmation. Because this study was based on publicly available genome assemblies, genome quality was assessed mainly using available NCBI Assembly metadata, BioSample metadata, and GenBank files. Assembly level, genome size, GC content, N50, and contig number were used as the main quality-control indicators. Genomes with sizes clearly outside the common genome-size range of *K. pneumoniae*, abnormal GC content, excessive contig numbers, low N50 values, low assembly levels, or incomplete metadata were manually reviewed together with their source information, species annotation, and usability for downstream analyses. In this study, approximately 4.5–6.5 Mb was used as the reference genome-size range for candidate *K. pneumoniae* genomes. Genomes with assembly quality considered insufficient for comparative genomic analysis were excluded from the final dataset.

To improve the transparency and reproducibility of dataset selection, the genome-screening workflow was further clarified. On 25 October 2025, an initial search of the NCBI Assembly database retrieved 135,380 *K. pneumoniae*-associated genome records. These records were then screened using bovine-associated keywords based on the host, isolation source, sample title, BioSample description, and GenBank source fields, followed by manual verification. Duplicate records were removed according to assembly accession, BioSample accession, assembly name, and strain name. For records with conflicting metadata across different fields or incomplete source information, the host and isolation source fields in BioSample were used as the primary criteria, together with the sample title, sample description, and GenBank source information for manual review.

Species confirmation was performed using FastANI v1.34. Genomes annotated as *K. pneumoniae* in the NCBI taxonomy system were first selected, and the average nucleotide identity (ANI) between each candidate genome and the reference genome *K. pneumoniae* subsp. pneumoniae MGH 78578 (RefSeq assembly accession: GCF_000016305.1) was then calculated. Only genomes with ANI values ≥95% and taxonomic results consistent with *K. pneumoniae* were retained. Records with inconsistent species annotations, ANI values below the species-level threshold, or higher similarity to other closely related *Klebsiella* species were manually reviewed; records that could not be confidently assigned to *K. pneumoniae* were excluded.

After source screening, duplicate removal, assembly-quality assessment, and species confirmation, 1291 bovine-associated *K. pneumoniae* genomes were retained for downstream analyses. These genomes were included in MLST typing, CARD-based antimicrobial resistance gene analysis, VFDB-based virulence gene analysis, PlasmidFinder-based plasmid replicon analysis, and Prokka/Roary-based core-genome and pangenome analyses. Roary analysis identified 46,325 gene clusters in the final dataset. A conservative strategy was applied to incomplete, ambiguous, or uncertain results generated during downstream analyses: genomes with incomplete MLST allelic profiles or profiles that could not be assigned to known sequence types were labeled as “unknown” or “undetermined”; antimicrobial resistance genes, virulence-associated genes, and plasmid replicon hits below the predefined identity or coverage thresholds were excluded, and results that could not be clearly classified were not forced into any category.

### 2.3. Molecular Typing

Molecular typing was performed using mlst v2.23.0 with the PubMLST/Pasteur seven-locus MLST scheme for *K. pneumoniae*. The analyzed loci included *rpoB*, *gapA*, *mdh*, *pgi*, *phoE*, *infB*, and *tonB*. Genome assembly sequence files downloaded from NCBI (genomic FASTA, .fna) were used as input. The software automatically assigned allele profiles and sequence types (STs) based on the allele sequences of the seven housekeeping genes. Genomes with complete seven-locus allele profiles that matched the database were assigned to the corresponding STs. Genomes with incomplete allele profiles, ambiguous matches, or profiles that could not be matched to known STs were labeled as unknown or undetermined and were not forcibly assigned to any ST. The resulting ST profiles were used to summarize the genetic diversity of bovine-associated *K. pneumoniae*.

### 2.4. Antimicrobial Resistance Gene Analysis

Antimicrobial resistance-associated genes were detected using ABRicate v1.0.1 in combination with the Comprehensive Antibiotic Resistance Database (CARD v4.0.1). Genome assembly sequence files in FASTA format (.fna) were used as input. Gene matches were retained only when both sequence identity and coverage were ≥80%; hits below these thresholds were excluded from subsequent statistical analyses. The detected resistance-associated genes were classified according to CARD annotations by antimicrobial category and resistance mechanism, including β-lactams, aminoglycosides, fosfomycins, quinolones, tetracyclines, sulfonamides, macrolides, phenicols, trimethoprim, rifampicin, polymyxins, disinfectant tolerance-associated genes, and multidrug resistance-associated determinants. For identical resistance-associated genes detected repeatedly within the same genome, gene-level deduplication was performed when calculating genome-level prevalence, whereas independent hits were retained when calculating the total number of matches.

It should be noted that this study used CARD-based genomic screening to identify antimicrobial resistance-associated genes or potential resistance determinants, and the results should not be interpreted as evidence of phenotypic resistance. To avoid overinterpretation, acquired or clinically important resistance-associated genes, such as *bla*, *tet*, *sul*, *qnr*, *mcr*, and aminoglycoside-modifying enzyme genes, were distinguished in the Results and Discussion sections from efflux pump-, membrane transport-, and permeability-associated determinants that may represent intrinsic or chromosomally conserved backgrounds, such as *oqxA*/*oqxB*, *acrA*, *KpnE*/*KpnF*/*KpnG*, and *OmpK37*. All genome-based predictions require further validation through phenotypic antimicrobial susceptibility testing, gene expression analysis, or functional assays.

### 2.5. Virulence Gene Analysis

Virulence-associated genes were screened using ABRicate v1.0.1 in combination with the Virulence Factor Database (VFDB; downloaded on 25 October 2025). Genome assembly sequence files (genomic FASTA, .fna) were used as input. Gene hits were retained only when sequence identity was ≥80% and coverage was ≥80%; hits below this threshold were excluded from downstream statistics. Detected virulence-associated genes were functionally classified according to VFDB annotations, including adhesion-associated factors, iron acquisition systems, siderophore-associated genes, and toxin-related determinants. For repeated hits of the same virulence-associated gene within a single genome, gene-level deduplication was performed for prevalence calculations. Hits that could not be clearly classified or were considered low-quality matches were labeled as not determined and were not included in the main statistical analyses.

### 2.6. Plasmid Replicon Analysis

Plasmid replicons were detected using ABRicate v1.0.1 in combination with the PlasmidFinder database and were classified according to the replicon nomenclature of PlasmidFinder v2.1.6. Genome assembly sequence files (genomic FASTA, .fna) were used as input. Replicon hits were retained only when sequence identity was ≥80% and coverage was ≥80%; hits below these thresholds were excluded from downstream statistics. The detected plasmid replicons were classified according to incompatibility groups, including IncF, IncHI, IncL/M, IncN, IncR, IncX, and IncY replicons; Col-type plasmid-associated replicons; and *repA* family replication-associated markers. For repeated hits of the same replicon within a single genome, deduplication was performed at the replicon-type level for genome-level prevalence calculations, whereas independent hits were retained when calculating the total number of replicon instances. Because this study was based on replicon detection, the presence of a specific replicon was interpreted only as evidence of a plasmid-associated genomic background and could not directly demonstrate physical co-localization between antimicrobial resistance genes and plasmid backbones.

It should be noted that PlasmidFinder-based replicon detection was used primarily to identify potential plasmid replicon types present in the genomes. Because contig-level co-localization analysis, complete plasmid reconstruction, long-read sequencing, and conjugation assays were not performed in this study, we could not determine whether antimicrobial resistance genes and specific plasmid replicons were located on the same contig, the same complete plasmid, or the same transferable genetic unit. Therefore, plasmid replicon-related results in this study reflect only plasmid replicon-associated genomic backgrounds or genome-level co-detection patterns with resistance genes, rather than direct evidence of plasmid localization of resistance genes or plasmid-mediated transfer.

### 2.7. Bioinformatics Tools, Database Versions, and Parameters

All bioinformatics analyses in this study were performed using publicly available genome assembly data, and raw sequencing reads were not used. The primary input files for sequence-based analyses were genome assembly sequence files downloaded from NCBI (genomic FASTA, .fna). GenBank flat files (.gbff), NCBI Assembly metadata, and BioSample metadata were used for annotation verification and metadata extraction.

Genome quality control was conducted mainly using NCBI Assembly metadata, BioSample metadata, and GenBank files. Assembly level, genome size, GC content, N50, and contig number were used to preliminarily assess assembly continuity and overall assembly quality. Genome size was evaluated with reference to the common genome-size range of *K. pneumoniae*, which is typically approximately 4.5–6.5 Mb. Candidate genomes with sizes clearly below or above this range, abnormal GC content, excessive contig numbers, low N50 values, low assembly levels, or incomplete metadata were manually reviewed in combination with species annotation, metadata completeness, and usability for downstream analyses. Molecular typing was performed using mlst v2.23.0 with the PubMLST/Pasteur seven-locus MLST scheme for *K. pneumoniae*. Antimicrobial resistance genes were detected using ABRicate v1.0.1 together with CARD v4.0.1. Virulence-associated genes were screened using ABRicate v1.0.1 together with the VFDB database. Plasmid replicons were detected using ABRicate v1.0.1 together with the PlasmidFinder database and were classified according to the replicon nomenclature of PlasmidFinder v2.1.6. The accession numbers, source metadata, and main genomic feature profiles of the genomes included in the final dataset are provided in Table S1.

Core-genome phylogenetic analysis and pangenome analysis were performed using genome annotation files as input. Pangenome analysis was conducted using Roary v3.13.0, with the protein sequence clustering threshold set at 95%. Based on the Roary output, gene clusters were classified into core genes, soft-core genes, shell genes, and cloud genes. Core genes were defined as gene clusters present in the vast majority of genomes, whereas soft-core, shell, and cloud genes represented accessory gene clusters detected across different frequency ranges. The core-gene alignment was used to construct the phylogenetic tree with FastTree v2.1.11, and the tree was visualized using iTOL v6. This analysis was used to complement the molecular typing results and to assess the genomic diversity of bovine-associated *K. pneumoniae* from both core-genome and pangenome perspectives.

Unless otherwise stated, gene hits in ABRicate analyses were filtered using a sequence identity threshold of ≥80% and a coverage threshold of ≥80%. Hits below these thresholds were excluded from downstream statistics. For repeated hits of the same antimicrobial resistance gene, virulence-associated gene, or plasmid replicon within a single genome, deduplication was performed at the gene or replicon-type level for genome-level prevalence calculations, whereas independent hits were retained when calculating total hit counts or the total number of replicon instances. All databases were accessed or updated on 25 October 2025. Genome accessions, assembly names, genome file names, NCBI download links, source metadata, and the results of MLST, antimicrobial resistance gene, VFDB, and PlasmidFinder analyses are provided in Table S1. Summaries of pangenome composition, antimicrobial resistance gene categories, virulence-associated gene categories, and plasmid replicons are provided in Table S2.

Custom Python scripts were used only for metadata parsing, keyword-based screening, duplicate removal, manual-curation table generation, and summary-table construction. They were not used to modify genome sequences or alter the outputs generated by MLST, ABRicate, Prokka, Roary, FastTree, or PlasmidFinder. The main input files for the scripts included NCBI Assembly metadata tables, BioSample metadata, GenBank flat files, and downstream result tables. The main outputs included the curated genome metadata table, bovine-associated genome list, duplicate-removal records, and summary tables for MLST, antimicrobial resistance-associated genes, virulence-associated genes, and plasmid replicons.

## 3. Results

### 3.1. Global Distribution of Publicly Available Bovine-Associated K. pneumoniae Genomes

**Figure 1:**
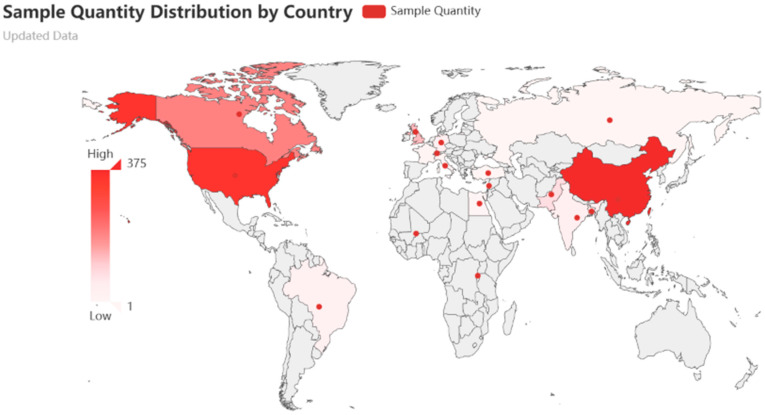
Geographical distribution of publicly available bovine-associated *K. pneumoniae* genomes.

The 1291 publicly available bovine-associated *K. pneumoniae* genomes included in this study were distributed across 18 countries, but their geographic distribution was markedly uneven. China and the United States were the two major countries of origin, contributing 375 genomes (29.05%) and 353 genomes (27.34%), respectively; together, they accounted for 56.39% of the total dataset. Canada contributed 195 genomes (15.10%), and Pakistan contributed 80 genomes (6.20%). A moderate number of genomes were obtained from India (39 genomes, 3.02%), Germany (32 genomes, 2.48%), Switzerland (30 genomes, 2.33%), and Brazil (26 genomes, 2.01%). Smaller numbers of genomes were obtained from Bangladesh (10 genomes), Italy (6 genomes), France (5 genomes), and Egypt (4 genomes), whereas Turkey, Rwanda, Russia, Lebanon, Guadeloupe, and other countries contributed only two genomes each. These results indicate that the current public dataset is geographically uneven and that publicly deposited bovine-associated *K. pneumoniae* genomes remain limited from several regions (Figure 1). It should be emphasized that the country-level distribution observed in this study reflects the availability and submission patterns of genome data in public databases, rather than direct differences in the true prevalence of bovine-associated *K. pneumoniae* across countries or regions. Differences in genome numbers among countries may be influenced by sampling intensity, sequencing project design, research priorities, public database submission practices, and metadata completeness.

### 3.2. Temporal Distribution of Publicly Available Bovine-Associated K. pneumoniae Genomes

**Figure 2:**
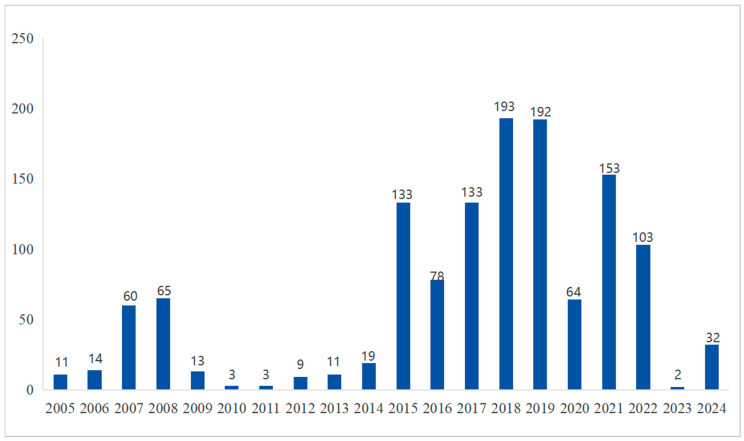
Temporal distribution of publicly available bovine-associated *K. pneumoniae* genomes.

The collection years of the 1291 genomes included in this study ranged from 2005 to 2024, and the number of publicly available genomes varied markedly across years (Figure 2). The number of genomes increased after 2015 and reached a peak in 2018, with 193 genomes, followed closely by 192 genomes in 2019. In addition, 133 genomes were recorded in both 2015 and 2017, and 153 genomes were recorded in 2021. In contrast, the number of genomes was relatively low from 2005 to 2014. Except for 60 genomes in 2007 and 65 genomes in 2008, most years during this period contained fewer than 20 genomes, with only three genomes recorded in both 2010 and 2011. After 2022, the number of publicly available genomes declined, reaching two genomes in 2023 and increasing to 32 genomes in 2024. Therefore, the temporal distribution observed in this study should be interpreted as changes in the availability of bovine-associated *K. pneumoniae* genomes in public databases, rather than direct changes in the true prevalence or disease incidence of this bacterium in cattle populations. Differences in the number of publicly available genomes among years may be influenced by the extent of sequencing technology application, the intensity of project-based surveillance, changes in research priorities, delays in data submission, and the completeness of database inclusion. Accordingly, comparisons of genome numbers across years should not be directly equated with epidemiological trends in disease occurrence.

### 3.3. MLST Typing Features and the Relative Predominance of ST107

Among the 1291 bovine-associated *K. pneumoniae* genomes included in this study, 256 distinct sequence types (STs) were identified, corresponding to 1089 genomes and accounting for 84.3% of the total dataset. The remaining 202 genomes were assigned as unknown STs, indicated as “-“, accounting for 15.7%. The ST distribution was broad and uneven. The top 20 STs ranked by frequency accounted for 28.9% of genomes with known STs and represented the major detected sequence types (Table 1). Among them, ST107 was the most common, comprising 125 genomes and accounting for 9.7% of the total dataset, which was markedly higher than the frequencies of other STs. ST37 ranked second, with 30 genomes (2.3%), while ST2324, ST219, and ST111 were each detected in 27 genomes, accounting for 2.1% each. The remaining top 20 STs occurred in 9 to 24 genomes, with proportions ranging from 0.7% to 1.9%, indicating the coexistence of multiple sequence types with relatively low individual prevalence.

However, MLST is based on only seven housekeeping genes and therefore cannot fully capture population differences at the whole-genome level. To address this limitation, we further integrated core-genome phylogenetic analysis and pangenome composition analysis to evaluate the genomic diversity of bovine-associated *K. pneumoniae* from a genome-wide perspective.

### 3.4. Core-Genome Phylogeny and Pangenome Diversity Analysis

**Figure 3:**
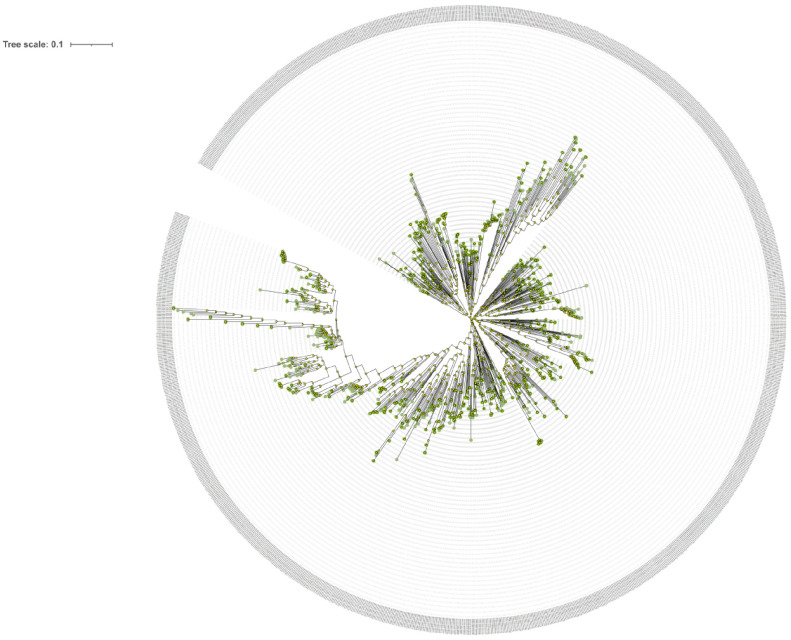
Core-genome phylogenetic tree of bovine-associated *K. pneumoniae*. The phylogenetic tree was constructed based on the core-genome alignment of 1291 bovine-associated *K. pneumoniae* genomes. The tree topology shows multiple branches and local clusters among different genomes, indicating substantial genetic diversity at the whole-genome level. The scale bar represents the number of nucleotide substitutions per site.

To overcome the limitations of relying solely on MLST, we further constructed a phylogenetic tree based on core-genome alignment and performed pangenome composition analysis of the 1291 bovine-associated *K. pneumoniae* genomes. The core-genome phylogenetic tree showed that bovine-associated *K. pneumoniae* genomes formed multiple distinct branches at the genome-wide level, with relatively long branch distances and several local clusters among different genomes. These results indicate substantial genomic heterogeneity within this bacterial population and suggest that it is not dominated by a single clonal lineage (Figure 3). This finding is consistent with the identification of 256 STs in the MLST analysis and further supports the high genetic diversity of bovine-associated *K. pneumoniae*.

**Figure 4:**
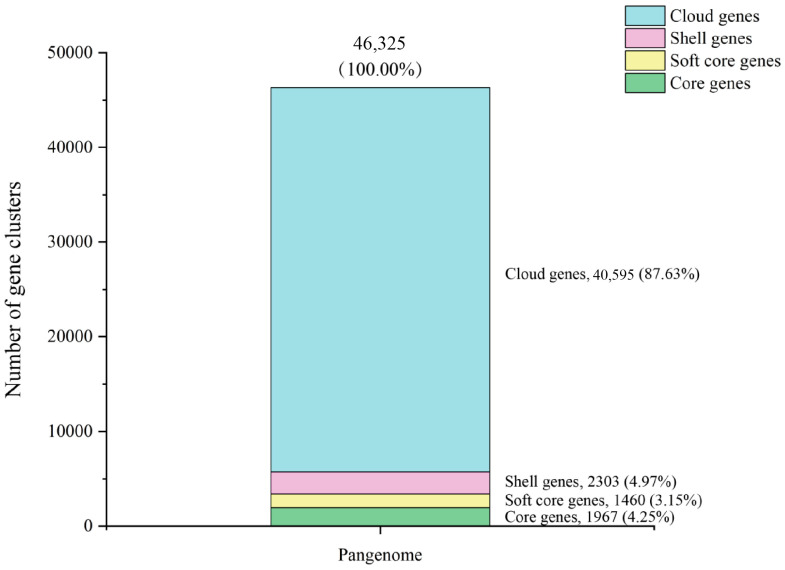
Pangenome composition of bovine-associated *K. pneumoniae*. Pangenome analysis of 1291 bovine-associated *K. pneumoniae* genomes identified 46,325 gene clusters. Cloud genes accounted for the largest proportion, with 40,595 gene clusters (87.63%), followed by shell genes with 2303 gene clusters (4.97%), soft-core genes with 1460 gene clusters (3.15%), and core genes with 1967 gene clusters (4.25%). The high proportion of cloud genes suggests an open pangenome structure and substantial accessory-gene diversity in bovine-associated *K. pneumoniae*.

Pangenome analysis identified a total of 46,325 gene clusters. Among them, 1967 were core genes, accounting for 4.25% of the total gene clusters; 1460 were soft-core genes, accounting for 3.15%; 2303 were shell genes, accounting for 4.97%; and 40,595 were cloud genes, accounting for 87.63% (Figure 4). Cloud genes represented the largest proportion of the pangenome, indicating a large accessory gene pool and a pronounced open pangenome structure in bovine-associated *K. pneumoniae*. The low proportion of core genes and the high proportion of cloud genes suggest marked differences in accessory gene composition among bovine-associated *K. pneumoniae* genomes from different sources, countries, and years. These differences may be associated with gene acquisition, gene loss, mobile genetic elements, and ecological niche adaptation. Overall, MLST provides useful information on the distribution of major sequence types, whereas core-genome phylogenetic and pangenome analyses further reveal the genetic diversity of bovine-associated *K. pneumoniae* at the whole-genome level.

### 3.5. Antimicrobial Resistance Gene Profiles of Bovine-Associated K. pneumoniae

**Figure 5:**
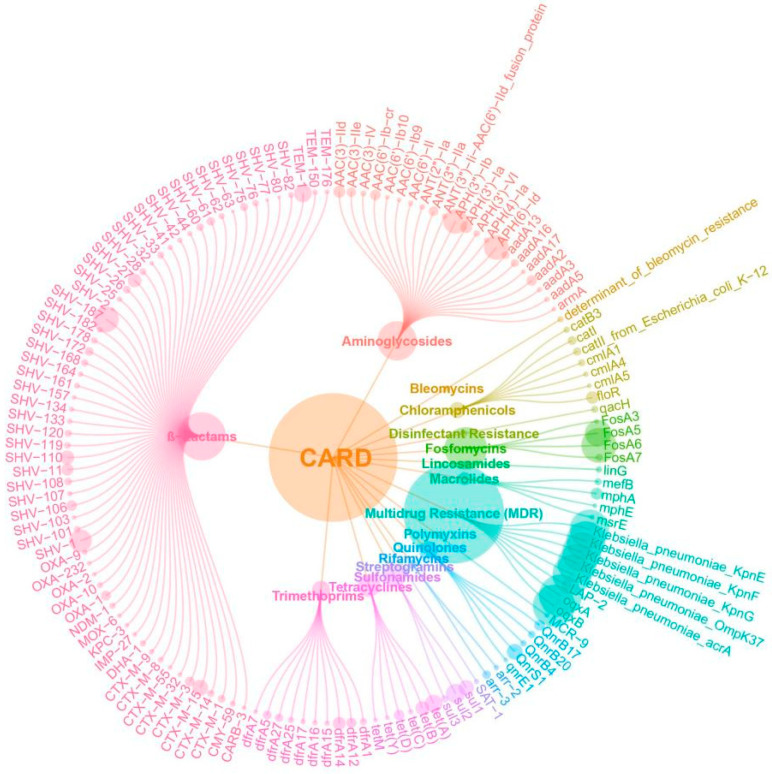
Circular network showing the distribution of antimicrobial resistance-associated genes and their corresponding antimicrobial categories in bovine-associated *K. pneumoniae*. Detailed gene-level information corresponding to this CARD-based circular network is provided in Table S3.

CARD was used to screen antimicrobial resistance-associated determinants in bovine-associated *K. pneumoniae* genomes. A total of 138 resistance-associated genes or potential resistance determinants were detected across 16 antimicrobial categories, with marked variation in their detection frequencies (Figure 5). It should be emphasized that these results are based on genome-based predictions and reflect the presence of resistance-associated genes rather than actual phenotypic resistance. Based on gene function and genetic background, the detected determinants can be broadly divided into two categories. The first category includes acquired or clinically important resistance-associated genes, such as those related to β-lactamases, carbapenemases, aminoglycoside-modifying enzymes, sulfonamides, tetracyclines, quinolones, and polymyxins. The second category includes efflux pump-, membrane transport-, and permeability-associated determinants that may represent intrinsic or chromosomally conserved backgrounds. Here, the term “AMR potential” refers to the presence of antimicrobial resistance-associated genes or potential resistance determinants predicted from genome data and should not be interpreted as confirmed phenotypic antimicrobial resistance.

Among β-lactam resistance genes, the SHV family showed the highest genetic diversity, comprising 28 genotypes. The most frequently detected genotypes were *bla*SHV-187 (501 hits), *bla*SHV-1 (360 hits), *bla*SHV-110 (108 hits), and *bla*SHV-27 (104 hits). The TEM family was dominated by *bla*TEM-1 (196 hits), whereas *bla*TEM-150 (4 hits) and *bla*TEM-176 (2 hits) were detected at relatively low frequencies. Within the CTX-M family, *bla*CTX-M-15 (151 hits), *bla*CTX-M-3 (38 hits), and *bla*CTX-M-1 (19 hits) were the predominant genotypes. In addition, clinically important β-lactamase- and carbapenemase-associated genes were detected, including *bla*OXA-1 (40 hits), *bla*NDM-1 (8 hits), *bla*KPC-3 (2 hits), and *bla*IMP-27 (2 hits), as well as cephalosporinase-associated genes such as *bla*DHA-1 (8 hits) and *bla*CMY-59 (6 hits).

For aminoglycoside resistance genes, *APH(6)-Id* (447 hits) and *APH(3″)-Ib* (442 hits) were the most frequently detected, followed by *AAC(3)-IId* (71 hits), *APH(3′)-Ia* (67 hits), and *aadA2* (47 hits). Additional aminoglycoside-modifying enzyme genes, such as *armA* (2 hits) and *ANT(2″)-Ia* (5 hits), were also detected, covering the three typical resistance mechanisms mediated by phosphotransferases, acetyltransferases, and nucleotidyltransferases. Among multidrug resistance-associated determinants, efflux pump-associated genes, including *oqxA* (1530 hits) and *oqxB* (1493 hits), as well as membrane transport- or permeability-associated genes, including *KpnG* (1290 hits), *KpnE* (1280 hits), *acrA* (1279 hits), *KpnF* (1276 hits), and *OmpK37* (1268 hits), were detected at high frequencies. Unlike typical acquired resistance genes, these efflux pump- and membrane-associated determinants may represent relatively conserved chromosomal backgrounds or intrinsic resistance-associated mechanisms in *K. pneumoniae*. Therefore, their high detection frequency should be interpreted as the widespread presence of resistance-associated genomic backgrounds rather than direct evidence of acquired multidrug-resistant phenotypes. Their actual effects on antimicrobial susceptibility require further validation through phenotypic antimicrobial susceptibility testing, gene expression analysis, and functional assays.

For fosfomycin resistance-associated genes, *FosA6* (994 hits) and *FosA5* (518 hits) accounted for 98.9% of all hits in this category and were the most frequently detected fosfomycin resistance-associated genes in this dataset. Sulfonamide resistance-associated genes were mainly represented by *sul2* (248 hits), *sul1* (138 hits), and *sul3* (15 hits). Tetracycline resistance-associated genes were dominated by *tet(A)* (205 hits), *tet(B)* (101 hits), and *tet(D)* (47 hits). Quinolone resistance-associated genes mainly included *QnrS1* (151 hits), *QnrB17* (52 hits), and *QnrB20* (15 hits). Among phenicol resistance-associated genes, the most frequently detected genes were *floR* (81 hits), *catI* (28 hits), and *catII* (22 hits), originally described in *Escherichia coli* K-12. In addition, the polymyxin resistance-associated gene *mcr-9* (2 hits), macrolide resistance-associated genes *mphA* (70 hits) and *mefB* (13 hits), the rifampicin resistance-associated gene *arr-3* (25 hits), trimethoprim resistance-associated genes *dfrA14* (86 hits) and *dfrA12* (43 hits), and the disinfectant tolerance-associated gene *qacH* (13 hits) were also detected. These results indicate that bovine-associated *K. pneumoniae* genomes harbor diverse antimicrobial resistance-associated determinants related to clinically important antibiotics and disinfectant tolerance. However, the corresponding resistance phenotypes still require confirmation by phenotypic antimicrobial susceptibility testing. To facilitate the interpretation of the dense gene-level information shown in Figure 5, the antimicrobial categories, detected counts, numbers of positive genomes, proportions of positive genomes, interpretation categories, and cautious notes for all CARD-detected antimicrobial resistance-associated genes and potential resistance determinants are provided in Table S3.

### 3.6. Virulence-Associated Gene Profiles of Bovine-Associated K. pneumoniae

**Figure 6:**
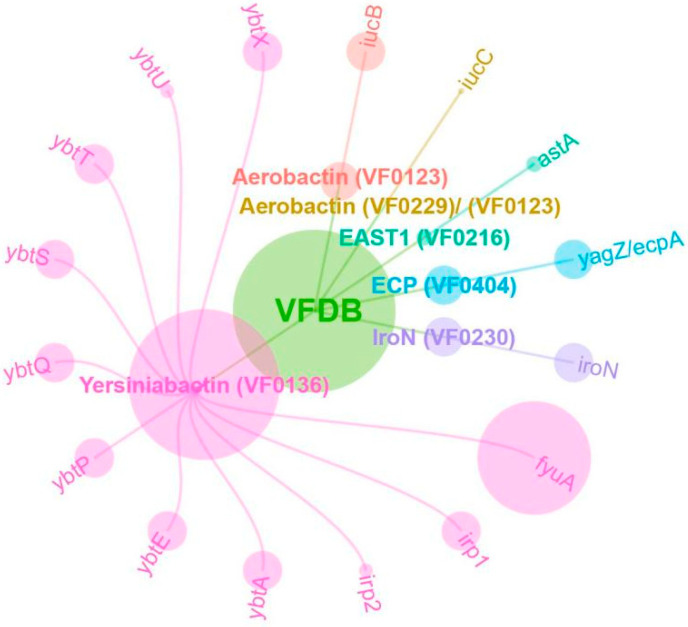
Circular network showing the virulence-associated genes detected in bovine-associated *K. pneumoniae* and their corresponding functional categories. Detailed gene-level information corresponding to this VFDB-based circular network is provided in Table S4.

Virulence-associated genes in bovine-associated *K. pneumoniae* were analyzed, and five virulence gene categories were identified, including the common pilus of *Escherichia coli* (ECP), yersiniabactin, aerobactin, salmochelin receptor-associated factors, and enterotoxin-associated virulence determinants. The detection numbers and proportions of these virulence-associated genes showed substantial heterogeneity (Figure 6).

Among the detected virulence-associated genes, the ECP-associated gene *yagZ/ecpA* was the most frequently detected determinant, being present in 996 genomes and accounting for 77.1% of the 1291 bovine-associated *K. pneumoniae* genomes. Yersiniabactin-associated genes showed variable detection frequencies: *ybtT* and *ybtE* were each detected in 100 genomes (7.7%); *ybtS*, *ybtQ*, *ybtA*, and *fyuA* were each detected in 99 genomes (7.7%); *ybtX* and *ybtU* were each detected in 98 genomes (7.6%); *ybtP* and *irp2* were each detected in 97 genomes (7.5%); and *irp1* was detected in 95 genomes (7.4%). Among aerobactin-associated genes, *iucB* and *iucC* were each detected in 9 genomes (0.7%). The salmochelin receptor-associated gene *iroN* was detected in only 4 genomes (0.3%). Among enterotoxin-associated genes, only *astA* was identified and was present in 12 genomes (0.9%).

Overall, *yagZ*/*ecpA* was the most frequently detected virulence-associated gene in bovine-associated *K. pneumoniae*, whereas yersiniabactin-, aerobactin-, salmochelin-, and enterotoxin-associated genes were detected only in a relatively small proportion of genomes. These results indicate substantial heterogeneity in the virulence-associated genomic features of bovine-associated *K. pneumoniae*. To facilitate further interpretation of the gene-level information presented in Figure 6, all VFDB-detected virulence-associated genes, functional categories, numbers of positive genomes, proportions of positive genomes, functional annotations, and cautious notes are summarized in Table S4.

### 3.7. Plasmid Replicon Profiles and Their Potential Association with Antimicrobial Resistance Determinants

In this study, plasmid-associated markers in bovine-associated *K. pneumoniae* genomes were detected and classified into three major categories: Col-type plasmid-associated markers, Inc incompatibility group markers, and replication-associated markers. Among these categories, Inc incompatibility group markers represented the most frequently detected plasmid-associated category and included multiple families, such as IncF, IncHI, IncL/M, IncN, IncR, IncX, and IncY. Among these markers, IncFIB(K)_1_Kpn3 showed the highest detection frequency, with 1093 instances identified in 1089 genomes, suggesting that IncF family replicons are highly prevalent among bovine-associated *K. pneumoniae* genomes. Other commonly detected replicons included IncHI1B_1_pNDM-MAR (424 genomes), IncFII_1_pKP91 (228 genomes; 230 instances), IncFIA(HI1)_1_HI1 (134 genomes), IncFIB(Mar)_1_pNDM-Mar (124 genomes), and IncFIB(pKPHS1)_1_pKPHS1 (106 genomes). In addition, IncR_1 (77 genomes), IncL/M(pMU407)_1_pMU407 (56 genomes), IncN3_1 (40 genomes), and IncL/M(pOXA-48)_1_pOXA-48 (36 genomes) were also detected (Table 2). These results indicate that bovine-associated *K. pneumoniae* genomes harbor diverse plasmid replicon profiles, with IncF family replicons being the most common. Importantly, this study was based only on plasmid replicon detection and did not include contig-level co-localization analysis or complete plasmid reconstruction. Therefore, the presence of specific replicons indicates only a plasmid replicon-associated genomic background and does not demonstrate that specific antimicrobial resistance genes are located on these plasmid backbones or that they have plasmid-mediated transferability.

Replication-associated markers were mainly represented by the *repA* family. Among them, repA_1_pKPC-2 was detected in 15 genomes, RepA_1_pKPC-CAV1321 was detected in 7 genomes, and repA_2_pKPC-2 was detected in 3 genomes. Compared with the major IncF family replicons, these replication-associated markers were detected at relatively low frequencies.

Overall, IncF and IncHI family replicons were frequently detected in bovine-associated *K. pneumoniae* genomes, suggesting that these plasmid replicon-associated genomic backgrounds may show a certain level of co-occurrence with antimicrobial resistance determinants. However, complete plasmid reconstruction, contig-level co-localization analysis, long-read sequencing, and conjugation assays were not performed in this study. Therefore, these results cannot demonstrate physical co-localization between resistance genes and plasmid backbones, nor can they directly prove plasmid-mediated transfer of antimicrobial resistance genes. These findings should be interpreted as genome-level potential associations rather than functional or experimental evidence.

## 4. Discussion

Based on 1291 publicly available genomes, this study provides a global genome-level analysis of bovine-associated *K. pneumoniae*. By integrating genetic diversity, virulence-associated gene profiles, antimicrobial resistance determinants, and plasmid replicon-associated genomic backgrounds, our findings indicate that bovine-associated *K. pneumoniae* represents a genetically diverse bacterial population with heterogeneous virulence-associated features and multiple antimicrobial resistance determinants. These results support the biological importance of bovine-associated *K. pneumoniae* as a potential microbial reservoir at the animal–environment–human interface and highlight the value of genome-based surveillance within a One Health framework.

The publicly available bovine-associated *K. pneumoniae* genomes analyzed in this study showed a markedly uneven geographic distribution, with China and the United States together accounting for 56.39% of the dataset. This pattern should not be interpreted simply as a true difference in global prevalence because the availability of public genomic data is strongly influenced by sampling intensity, sequencing capacity, research priorities, and database submission practices [11,12]. As major cattle-producing countries with active surveillance and sequencing efforts, China and the United States may have contributed more bovine-associated genome data to public databases. In contrast, the small number of genomes from some countries may reflect limited sampling or underrepresentation in public repositories rather than a genuinely low prevalence of bovine-associated *K. pneumoniae* [13]. Therefore, although the geographic distribution observed in this study provides important information on the current landscape of publicly available genomes, it should be interpreted cautiously when inferring global epidemiological patterns. Future surveillance efforts should improve accuracy by implementing more balanced sampling across countries and production systems.

The number of publicly available bovine-associated *K. pneumoniae* genomes increased substantially after 2015 and peaked between 2018 and 2019. This temporal pattern likely reflects increased research attention to antimicrobial resistance, the broader adoption of whole-genome sequencing, and improved submission of genomic data to public databases, rather than directly indicating changes in disease incidence or bacterial prevalence [14,15]. The decline in genome numbers after 2022 should also be interpreted cautiously, as recent data may be affected by incomplete database deposition or delays in metadata release. Therefore, the temporal pattern observed in this study mainly reflects changes in genome availability and surveillance intensity. To accurately assess the true dynamics of antimicrobial resistance in bovine-associated *K. pneumoniae*, genomic data should be integrated with antimicrobial usage records, farm-level sampling data, and phenotypic antimicrobial susceptibility testing results.

A total of 256 STs were identified in this study, indicating substantial genetic diversity among bovine-associated *K. pneumoniae* genomes. ST107 was the most common sequence type, accounting for 9.7% of all included genomes. The predominance of ST107 suggests that this lineage may be widely distributed among bovine-associated *K. pneumoniae* populations. However, whether ST107 has specific colonization advantages, enhanced environmental tolerance, or increased adaptation to bovine-associated niches requires further experimental validation [6,14]. In addition to ST107, multiple other STs, including ST37, ST2324, ST219, and ST111, were also detected, indicating that bovine-associated *K. pneumoniae* is not dominated by a single lineage but instead comprises diverse genetic backgrounds. Together with global reports on the convergence of hypervirulence and carbapenem resistance and molecular epidemiological evidence from China [13,15], previous studies have reported the mobilization of virulence plasmids in hypervirulent *K. pneumoniae* [16], the emergence of hypermucoviscous colistin-resistant high-risk convergent clones [17], carbapenem-resistant virulence plasmid-harboring isolates [18], IncX3 plasmid-associated *bla*NDM transmission [19], persistence of *K. pneumoniae* ST258 within human neutrophils [20], and additional clinically important lineages or outbreak-associated phenotypes [21,22]. These findings suggest potential genetic overlap, genomic plasticity, and adaptive capacity across different ecological hosts. In addition, 15.7% of the genomes could not be assigned to known STs, which may be associated with incomplete allele profiles, suboptimal genome assembly quality, or insufficient representation of bovine-associated lineages in current MLST databases. Therefore, ST107 may serve as a useful genomic marker for monitoring bovine-associated *K. pneumoniae*, but its epidemiological and functional significance should be interpreted with caution.

It should be noted that MLST is based on only seven housekeeping genes and cannot fully represent genome-wide population structure. Therefore, this study further incorporated core-genome phylogenetic analysis and pangenome composition analysis to complement molecular typing. The core-genome phylogenetic tree showed that the 1291 bovine-associated *K. pneumoniae* genomes formed multiple branches and local clusters, indicating substantial genome-wide genetic heterogeneity. Pangenome analysis further revealed 46,325 gene clusters in this dataset, with cloud genes accounting for 87.63% and core genes accounting for only 4.25%. These findings suggest that bovine-associated *K. pneumoniae* has a pronounced open pangenome structure, and its genomic variation is reflected not only in ST distribution but also in the high diversity of the accessory gene pool. Therefore, the related findings in this study are described as “genetic diversity” or “genomic diversity”. Therefore, these findings are interpreted as evidence of genetic and genomic diversity rather than as a high-resolution population-structure inference based solely on MLST.

The detection of 138 antimicrobial resistance genes belonging to 16 antimicrobial categories indicates that bovine-associated *K. pneumoniae* genomes harbor diverse antimicrobial resistance determinants. Among these categories, multidrug resistance-associated genes showed the highest overall detection frequency. The high prevalence of *oqxA/oqxB* and membrane protein-associated genes suggests that efflux pump-related and permeability-associated determinants may contribute to the antimicrobial resistance potential of bovine-associated *K. pneumoniae* [23,24].

Among β-lactam resistance genes, the SHV family showed high diversity, with *bla*SHV-187, *bla*SHV-1, *bla*SHV-110, and *bla*SHV-27 being frequently detected [25]. Clinically important carbapenemase-associated genes, including *bla*NDM-1, *bla*KPC-3, and *bla*IMP-27, were detected at relatively low frequencies. Although these genes were limited in distribution within this dataset, their presence in bovine-associated genomes remains noteworthy because carbapenemase genes are important antimicrobial resistance determinants in both clinical and One Health contexts [26]. Fosfomycin resistance-associated genes, mainly *FosA6* and *FosA5*, were also commonly detected, whereas the disinfectant tolerance-associated gene *qacH* was identified in only a small number of genomes.

These findings suggest that bovine-associated *K. pneumoniae* genomes harbor both acquired or clinically important resistance-associated genes and efflux pump-, membrane transport-, and permeability-associated determinants that may represent intrinsic or chromosomally conserved backgrounds. The former, such as *bla*NDM, *bla*KPC, *bla*CTX-M, tet, s*ul*, *qnr*, and *mcr*, are more suitable as priority targets for monitoring potential acquired resistance risks. In contrast, the latter, including *oqxA/oqxB*, *acrA*, *KpnE/KpnF/KpnG*, and *OmpK37*, should be interpreted as auxiliary indicators of resistance-associated genomic backgrounds or intrinsic resistance potential. Because corresponding phenotypic antimicrobial susceptibility data and gene expression data were not available in this study, the detection of resistance-associated genes and potential resistance determinants cannot be directly inferred as evidence of phenotypic resistance. Future studies integrating antimicrobial susceptibility testing, transcriptional analysis, gene localization analysis, and functional validation are required to further clarify the contribution of these resistance-associated determinants to actual resistance phenotypes [27].

Virulence-associated gene analysis revealed a heterogeneous distribution of virulence determinants among bovine-associated *K. pneumoniae* genomes. The ECP-associated gene *yagZ/ecpA* was the most frequently detected virulence-associated determinant, being present in 996 of the 1291 genomes (77.1%). This finding suggests that ECP-associated adhesion functions may represent a common virulence-associated genomic feature of bovine-associated *K. pneumoniae*. In contrast, yersiniabactin-associated genes were detected in only a smaller proportion of genomes, with *fyuA* present in 99 genomes (7.7%). The *fyuA*-encoded yersiniabactin receptor is involved in siderophore-mediated iron acquisition and may contribute to bacterial adaptation in iron-limited host environments [28,29]. However, because this study was based on genome mining rather than gene expression analysis or functional experiments, the roles of *yagZ/ecpA*, *fyuA*, and other virulence-associated genes in colonization, virulence, or host adaptation among bovine-associated genomes should be interpreted cautiously. Aerobactin-, salmochelin-, and enterotoxin-associated genes were detected at relatively low frequencies, indicating marked differences in virulence-associated features among bovine-associated *K. pneumoniae* genomes. Further phenotypic studies and infection model experiments are needed to clarify the contribution of these virulence determinants to pathogenicity [23,30].

The coexistence of adhesion-associated determinants, siderophore-associated genes, antimicrobial resistance genes, and plasmid replicons suggests that bovine-associated *K. pneumoniae* may harbor genomic backgrounds associated with both pathogenicity and antimicrobial resistance. However, because this study was based on genome mining, the functional contributions of these determinants to colonization, infection, or transmission require further experimental validation.

Plasmid replicon analysis showed that IncF-family replicons were frequently detected in bovine-associated *K. pneumoniae* genomes. Among them, IncFIB(K)_1_Kpn3 was the most common, suggesting that IncF-family replicon-associated genetic backgrounds are widespread in this bacterial population. Other replicons, including IncHI1B_1_pNDM-MAR, IncFII_1_pKP91, IncFIA(HI1)_1_HI1, and IncFIB(Mar)_1_pNDM-Mar, were also detected in a subset of genomes, reflecting the diversity of plasmid replicon profiles among bovine-associated *K. pneumoniae*. Previous studies have reported that replicons such as IncF, IncHI, and IncL/M are commonly found in antimicrobial resistance-associated plasmid backgrounds in Enterobacteriaceae and may co-occur at the genomic level with β-lactamase- or carbapenemase-associated genes [4,27,31,32, 33, 34]. In addition, comparative plasmid studies have shown that *mcr-1*-bearing plasmids may differ in invasion and persistence capacity between *E. coli* and *K. pneumoniae*, highlighting the host-dependent nature of plasmid maintenance and the need for additional functional validation beyond replicon detection [35]. However, because this study was based on PlasmidFinder replicon screening and did not include contig-level co-localization analysis, complete plasmid reconstruction, long-read sequencing, or conjugation assays, these findings should be interpreted as genome-level co-detection patterns or potential plasmid-associated genomic backgrounds rather than direct evidence of plasmid-mediated carriage, localization, or transfer of antimicrobial resistance genes. However, because this study was based on PlasmidFinder replicon screening and did not include contig-level co-localization analysis, complete plasmid reconstruction, long-read sequencing, or conjugation assays, these findings should be interpreted as genome-level co-detection patterns or potential plasmid-associated genomic backgrounds rather than direct evidence of plasmid-mediated carriage, localization, or transfer of antimicrobial resistance genes. Therefore, IncF, IncHI, IncL/M, IncN, and IncR replicons may serve as potential genomic indicators for monitoring plasmid-associated resistance risk in bovine-associated *K. pneumoniae*, but their genetic location, mobility, and transferability require further validation through long-read sequencing, hybrid assembly, plasmid reconstruction, and functional transfer assays [33,34].

### 4.1. Implications of This Study for the Prevention and Treatment of Bovine-Associated K. pneumoniae

Based on 1291 publicly available bovine-associated *K. pneumoniae* genomes, this study systematically revealed the genetic diversity, resistance-associated genes, virulence-associated genes, and plasmid replicon backgrounds of this bacterial population. Although phenotypic antimicrobial susceptibility testing, infection models, and vaccine-protection assays were not performed in this study, the genomic features identified here may still provide useful references for risk surveillance, precision antimicrobial use, and the screening of candidate prevention and control targets. The major candidate genomic targets identified in this study and their potential relevance for the prevention and control of bovine-associated *K. pneumoniae* are summarized in Table 3.

From a therapeutic perspective, the detection of β-lactamase- or carbapenemase-associated genes, including *bla*SHV, *bla*TEM, *bla*CTX-M, *bla*NDM, *bla*KPC, *bla*OXA, and *bla*IMP, suggests that some isolates may possess complex resistance-associated genetic backgrounds. Therefore, clinical prevention and treatment should integrate bacterial isolation, antimicrobial susceptibility testing, and resistance gene detection, rather than relying solely on empirical antimicrobial use [3,14]. From a preventive perspective, *yagZ/ecpA* and iron acquisition-associated genes, such as *fyuA*, *ybt*, *iuc*, and *iro*, may represent important targets for studies on colonization, invasion, and candidate antigens. Previous studies have shown that siderophore receptor proteins and outer membrane-associated proteins of *K. pneumoniae* have potential value in vaccine development or immune intervention strategies [36,37]. Plasmid replicons such as IncF, IncHI, IncL/M, IncN, and IncR may serve as potential surveillance markers for plasmid-associated antimicrobial resistance dissemination risk; however, their transferability still requires further validation through long-read sequencing, plasmid assembly, and conjugation assays [33].

### 4.2. Potential Impacts of Resistance and Virulence Features on Cattle Production

Bovine-associated *K. pneumoniae* is an important environmental mastitis pathogen and may originate from bedding materials, manure, water, soil, farm environments, and teat surfaces. Infection is more likely to occur under poor environmental hygiene, inadequate bedding management, or insufficient milking sanitation [14]. Its impact on cattle production is mainly reflected in mastitis occurrence, increased treatment difficulty, reduced milk yield, decreased milk quality, discarded milk, increased medication costs, and a higher risk of culling. The multiple categories of antimicrobial resistance-associated genes and potential resistance determinants detected in this study suggest that some isolates may possess complex resistance-associated genetic backgrounds. In particular, the coexistence of β-lactamase-associated genes, multidrug efflux pump-associated genes, and plasmid replicons may increase the complexity of empirical treatment selection and highlights the need to strengthen resistance surveillance and antimicrobial susceptibility testing-guided prudent antimicrobial use [23]. Virulence-associated genes may contribute to mammary epithelial adhesion, adaptation to iron-limited environments, immune evasion, and tissue colonization, thereby increasing the risk of persistent or recurrent infection [28,29]. Therefore, control strategies at the farm level should extend beyond clinical treatment alone and integrate environmental hygiene, bedding management, teat disinfection, rational antimicrobial use, resistance monitoring, and tracking of high-risk strains. The potential impacts of these resistance- and virulence-associated genomic features on cattle production and their corresponding management implications are summarized in Table 4. It should be noted that the findings of this study are based on genome-based predictions, and their actual effects on clinical treatment outcomes and production performance require further validation through antimicrobial susceptibility testing, infection models, and farm-level epidemiological data.

### 4.3. Microbial Associations and Microbiota-Oriented Control Implications

In addition to the antimicrobial resistance- and virulence-associated determinants of the pathogen itself, mammary, intestinal, and farm-environment microbiota may also influence the colonization, persistence, and recurrence of bovine-associated *K. pneumoniae* (Table 5). Previous milk microbiota analysis showed that cows with recurrent *Klebsiella*-associated mastitis harbored increased levels of intestinal-associated bacteria, such as Ruminococcaceae, Faecalibacterium, and Bacteroidales, suggesting possible fecal contamination and highlighting the importance of bedding, manure, teat, and environmental hygiene management [38]. From a microbiota-oriented intervention perspective, some lactic acid bacteria or probiotic candidates may have potential value. For example, *W. cibaria* SDS2.1 was reported to inhibit *K. pneumoniae* growth, reduce bacterial adhesion and invasion, and alleviate mammary inflammation and tissue damage in experimental models [39]. Therefore, the prevention of bovine-associated *K. pneumoniae* should not focus solely on the pathogen itself but should integrate pathogen surveillance, antimicrobial resistance and virulence monitoring, farm environmental control, and microbiota-based intervention strategies. Future studies combining metagenomics, bacterial isolation, functional microbiota analysis, and controlled animal trials are needed to identify protective microbial markers and evaluate their practical value in reducing the risk of *Klebsiella*-associated mastitis.

### 4.4. Limitations

This study has several limitations. First, it was entirely based on publicly available genome assemblies retrieved from the NCBI public database and did not use a unified sampling design. Therefore, differences in genome numbers among countries, years, and sample types may be influenced by sampling intensity, sequencing projects, research priorities, public database submission practices, and metadata completeness and should not be directly interpreted as true differences in prevalence or disease incidence. Second, genome quality assessment mainly relied on existing assembly metadata and basic assembly metrics. Although genome size, GC content, N50 value, contig number, and FastANI-based species confirmation were used for dataset screening, differences in sequencing and assembly workflows among genomes from different sources may still affect downstream analyses. Third, the antimicrobial resistance and virulence features described in this study were mainly inferred from genome-based predictions. The detection of resistance-associated genes, virulence-associated genes, or plasmid replicons does not necessarily indicate actual phenotypic resistance, pathogenic potential, or transferability. In particular, PlasmidFinder-based replicon detection cannot demonstrate the physical co-localization of resistance genes with specific plasmid backbones, nor can it directly support plasmid-mediated horizontal transfer. Future studies integrating standardized sampling, phenotypic antimicrobial susceptibility testing, long-read sequencing, plasmid reconstruction, conjugation assays, and infection models are needed to further validate these findings.

## 5. Conclusions

Based on 1,291 publicly available bovine-associated *K. pneumoniae* genomes, this study systematically analyzed the genetic diversity, virulence-associated genes, antimicrobial resistance-associated determinants, and plasmid replicon profiles of this bacterial population. MLST identified 256 sequence types, while core-genome phylogenetic and pangenome analyses further revealed substantial whole-genome heterogeneity and an open pangenome structure. Virulence-associated genes were unevenly distributed, with *yagZ*/*ecpA* being widely detected, whereas yersiniabactin-, aerobactin-, salmochelin-, and enterotoxin-associated genes were present only in a subset of genomes. The detection of multiple categories of antimicrobial resistance-associated genes and potential resistance determinants suggests that bovine-associated *K. pneumoniae* may serve as a potential reservoir of resistance-associated genetic determinants at the genomic level; however, this AMR potential should not be interpreted as confirmed phenotypic resistance without antimicrobial susceptibility testing. IncF-family plasmid replicons, particularly IncFIB(K)_1_Kpn3, were frequently detected, indicating widespread plasmid replicon-associated genomic backgrounds. However, replicon detection alone cannot confirm plasmid localization or transferability of resistance genes, and further validation using long-read sequencing and functional experiments is required. From an applied perspective, *yagZ*/*ecpA*, fyuA and other iron acquisition-associated genes, β-lactamase-associated genes, multidrug efflux pump-associated genes, and IncF/IncHI plasmid replicons may serve as key targets for future risk surveillance and candidate control-target screening. Overall, this study provides genomic insights for the surveillance of bovine-associated *K. pneumoniae*, antimicrobial resistance risk assessment, and integrated control strategies within a One Health framework.

## Figures and Tables

**Table 1:** Top 20 sequence types of bovine-associated *K. pneumoniae* and their housekeeping gene profiles.

Sequence Type (ST)	*gapA*	*infB*	*mdh*	*pgi*	*phoE*	*rpoB*	*tonB*	Count	Proportion
ST107	2	1	2	17	27	1	39	125	9.68%
ST37	2	9	2	1	13	1	16	30	2.32%
ST2324	2	1	1	1	16	4	4	27	2.09%
ST219	2	1	2	3	27	1	39	27	2.09%
ST111	2	1	5	1	17	4	42	27	2.09%
ST294	2	1	2	1	4	4	87	24	1.86%
ST2640	2	1	1	1	1	4	13	21	1.63%
ST661	4	3	1	36	9	10	14	19	1.47%
ST76	4	1	1	1	21	1	35	16	1.24%
ST5729	2	1	2	17	27	1	1	15	1.16%
ST43	2	6	1	5	11	1	15	15	1.16%
ST14	1	6	1	1	1	1	1	14	1.08%
ST234	2	1	2	1	7	1	24	12	0.93%
ST29	2	3	2	2	6	4	4	11	0.85%
ST5370	2	1	195	1	9	1	13	10	0.77%
ST2854	3	1	1	1	1	1	31	10	0.77%
ST716	71	1	1	2	16	4	164	10	0.77%
ST45	2	1	1	6	7	1	12	10	0.77%
ST229	4	6	1	1	8	1	56	9	0.70%
ST199	4	34	1	1	21	1	35	9	0.70%

**Table 2:** Detection results and cautious genomic-level interpretation of plasmid replicons and related markers in bovine-associated *K. pneumoniae*.

Core Classification Type	Gene	Count	Genomic-Level Interpretation and Cautious Statement
Col plasmid functional gene	*Col156_1*	9	A Col-type plasmid-associated marker detected at low frequency; its relationship with antimicrobial resistance genes was not determined in this study.
	*Col3M_1*	2	A low-frequency Col-type marker, suggesting a rare Col plasmid-associated genomic background in this dataset.
	*Col440I_1*	576	A frequently detected Col-type marker, indicating a common Col plasmid-associated background; however, its co-localization with specific AMR genes cannot be inferred from replicon detection alone.
	*Col440II_1*	61	A Col440-related marker detected in a subset of genomes; its functional association with AMR genes requires further structural validation.
	*ColE10_1*	4	A low-frequency Col-type marker detected in a small number of genomes; no direct AMR association can be concluded.
	*ColKP3_1*	2	A rare Col-type marker previously described in *K. pneumoniae*-related plasmid contexts; its biological significance in this dataset remains to be verified.
	*Col(MG828)_1*	12	A minor Col-type marker detected at low frequency; its potential association with specific plasmid structures requires further investigation.
	*ColpVC_1*	16	A Col-type marker detected in a limited number of genomes; this result should be interpreted as plasmid-marker detection rather than evidence of functional AMR linkage.
	*ColRNAI_1*	21	A Col plasmid replication-associated marker detected in a subset of genomes; its co-occurrence with AMR genes was not resolved at the plasmid-structure level.
Inc incompatibility group marker gene	*IncA/C2_1*	12	An IncA/C2-related replicon detected at low frequency; although this replicon type has been reported in AMR-associated plasmid backgrounds, no direct AMR gene co-localization was demonstrated here.
	*IncFIA(HI1)_1_HI1*	134	An IncFIA/IncHI1-associated replicon detected in this dataset; co-detection with other Inc-family replicons may indicate complex plasmid-associated genomic backgrounds, but not direct AMR gene localization.
	*IncFIB(K)_1_Kpn3*	1093	The most frequently detected IncF-family replicon in this dataset, suggesting that IncF-related plasmid-associated backgrounds are common in bovine-associated *K. pneumoniae* genomes; co-localization with AMR genes requires complete plasmid reconstruction or long-read sequencing.
	*IncFIB(Mar)_1_pNDM-Mar*	124	An IncFIB(Mar)-family replicon similar to previously reported pNDM-Mar-related plasmid backgrounds; its detection suggests a potential plasmid-associated genomic background, but does not prove that NDM or other AMR genes are located on the same plasmid.
	*IncFIB(pKPHS1)_1_pKPHS1*	106	A common IncFIB-family replicon detected in *K. pneumoniae*; its presence indicates a plasmid-associated marker, but its relationship with AMR genes remains unresolved.
	*IncFIB(pQil)_1_pQil*	2	A low-frequency IncFIB-family replicon; no direct functional or AMR-related conclusion can be drawn from its detection alone.
	*IncFII(pCRY)_1_pCRY*	2	A rare IncFII-family replicon detected in this dataset; potential linkage with AMR genes requires contig-level or complete-plasmid evidence.
	*IncFII(pCTU2)_1_pCTU2*	2	A low-frequency IncFII-family replicon; its co-localization with AMR determinants was not established in this study.
	*IncFII(pKPX1)*	11	An IncFII-family replicon previously reported in *K. pneumoniae* plasmid backgrounds; in this study, it should be interpreted only as a plasmid replicon marker.
	*IncFII(pMET)_1_pMET1*	2	A rare IncFII-family replicon; no direct association with specific AMR genes can be inferred without plasmid reconstruction.
	*IncFII_1*	2	A low-frequency IncFII-related marker with no resolved plasmid structure in this study; its biological significance remains uncertain.
	*IncFII_1_pKP91*	230	An IncFII-family replicon detected in multiple genomes; co-detection with IncF-family markers may indicate complex plasmid replicon profiles, but not direct AMR gene localization or transferability.
	*IncHI1B_1_pNDM-MAR*	424	An IncHI1B-family replicon similar to previously reported pNDM-MAR-related plasmid backgrounds; its detection suggests a potential plasmid-associated genomic background, but does not prove co-localization with NDM or other AMR genes.
	*IncHI2_1*	22	An IncHI2-family replicon detected in a subset of genomes; its potential association with AMR-related plasmid backgrounds requires further validation.
	*IncHI2A_1*	22	An IncHI2A-related marker detected at the same frequency as IncHI2_1; its relationship with AMR genes cannot be determined from replicon screening alone.
	*IncI1_1_Alpha*	9	A low-frequency IncI1-family replicon; although IncI1 plasmids have been reported in AMR-related contexts, this study did not demonstrate AMR gene co-localization.
	*IncL/M(pMU407)_1_pMU407*	56	An IncL/M-family replicon detected in a subset of genomes; it may indicate a potential plasmid-associated background, but direct linkage with β-lactam resistance genes was not resolved.
	*IncL/M(pOXA-48)_1_pOXA-48*	36	An IncL/M-family replicon similar to reported OXA-48-like plasmid backgrounds in Enterobacteriaceae; its detection does not prove that OXA-48-like genes are present on the same plasmid in this dataset.
	*IncL/M_1*	3	A rare IncL/M-family marker; its association with specific AMR genes or transferability requires further validation.
	*IncN2_1*	9	A low-frequency IncN-family subtype; its potential AMR association cannot be inferred without contig-level co-localization or complete plasmid evidence.
	*IncN3_1*	40	An IncN-family replicon detected in a subset of genomes; although IncN plasmids have been reported in AMR-related backgrounds, this study only supports genome-level replicon detection.
	*IncN_1*	31	An IncN-family replicon detected in a subset of genomes; its relationship with AMR determinants remains to be validated.
	*IncR_1*	77	An IncR-family replicon detected in multiple genomes; its presence suggests a potential plasmid-associated genomic background rather than direct evidence of AMR gene transfer.
	*IncX1_1*	5	A rare IncX-family replicon; no direct association with quinolone resistance genes can be concluded from this dataset.
	*IncX4_2*	8	A low-frequency IncX-family replicon; potential linkage with AMR genes or transferability requires further experimental and structural confirmation.
	*IncX5_2*	4	A rare IncX-family replicon; its co-localization with AMR genes was not demonstrated.
	*IncX6_1*	5	A low-frequency IncX-family replicon; its AMR-related significance remains uncertain and requires validation.
	*IncY_1*	15	An IncY-family replicon detected in a limited number of genomes; its relationship with tetracycline or other AMR genes cannot be inferred without additional evidence.
Replication-related gene (repA family)	*repA_1_pKPC-2*	15	A repA-family replication-associated marker similar to previously reported pKPC-related plasmid backgrounds; this study does not prove that KPC-related genes are located on the same plasmid.
	*repA_2_pKPC-2*	3	A low-frequency repA-family marker related to pKPC-type plasmid backgrounds; its functional significance and AMR gene co-localization require further validation.
	*RepA_1_pKPC-CAV1321*	7	A repA-family marker similar to reported pKPC-CAV1321-related plasmid backgrounds; complete plasmid reconstruction is required to determine its relationship with AMR genes.

**Table 3:** Candidate genomic targets and their potential relevance for the prevention and control of bovine-associated *K. pneumoniae*.

Target Category	Representative Genes/Markers	Findings in This Study	Potential Control Relevance	Future Validation
Adhesion-associated factor	*yagZ/ecpA*	Most frequently detected virulence-associated gene	Potential marker for colonization and mammary epithelial adhesion risk	Adhesion assays, gene expression analysis, mammary epithelial cell infection models
Iron acquisition/yersiniabactin system	*fyuA, ybtA, ybtS, ybtE, irp1, irp2*	Detected in a subset of genomes	May contribute to adaptation in iron-limited host environments and serve as candidate antigen or virulence marker	Antigen conservation analysis, immune protection assays, iron-limited growth assays
Aerobactin/salmochelin-associated factors	*iucB, iucC, iroN*	Detected at low frequencies	Associated with iron acquisition and potentially high-risk virulence backgrounds	Correlation with virulence phenotypes and animal infection models
β-lactam resistance genes	*bla*SHV*, bla*TEM, *bla*CTX-M, *bla*NDM, *bla*KPC, *bla*OXA, *bla*IMP	Multiple β-lactamase- or carbapenemase-associated genes were detected	Useful for antimicrobial resistance surveillance and prudent therapy; may indicate risk of treatment failure	Phenotypic antimicrobial susceptibility testing, gene expression analysis, plasmid localization
Multidrug efflux/membrane permeability-associated genes	*oqxA, oqxB, acrA, KpnE, KpnF, KpnG, OmpK37*	Frequently detected among multidrug resistance-associated determinants	Potential auxiliary indicators of resistance-associated genomic backgrounds; phenotypic effects require further validation	Efflux pump inhibition assays, transcriptomic validation, genotype–phenotype association analysis
Plasmid replicons	IncFIB(K), IncHI1B, IncFII, IncL/M, IncN, IncR	Diverse plasmid replicon backgrounds were detected	Potential markers for monitoring plasmid replicon-associated AMR risk; gene localization and transferability require further validation	Long-read sequencing, complete plasmid reconstruction, conjugation assays
Capsule- and outer membrane protein-associated antigens	*K-locus*, *wzi/wzc*, *OmpA*, *OmpK36*, *OmpK17*	Capsule typing and outer membrane protein antigen analysis were not systematically performed in this study	Important surface structures of *K. pneumoniae*; potential targets for vaccine development and molecular typing	K-locus typing, serotyping, protein expression analysis, immunogenicity evaluation

**Table 4:** Potential impacts of resistance- and virulence-associated genomic features on cattle production and management implications.

Genomic Feature	Representative Genes/Factors	Potential Impact on Cattle or Production	Management and Control Implications
β-lactam resistance-associated genes	*bla*SHV, *bla*TEM, *bla*CTX-M, *bla*NDM, *bla*KPC, *bla*OXA, *bla*IMP	May reduce β-lactam treatment efficacy and increase the risk of therapeutic failure	Combine antimicrobial susceptibility testing with treatment decisions and avoid empirical overuse
Multidrug resistance-associated mechanisms	*oqxA*, *oqxB*, *acrA*, *KpnE*, *KpnF*, *KpnG*, *OmpK37*	May indicate resistance-associated genomic backgrounds; actual effects on antimicrobial susceptibility require phenotypic validation	Establish farm-level AMR gene surveillance and antimicrobial use records
Plasmid replicon-associated backgrounds	IncF, IncHI, IncL/M, IncN, IncR	May indicate potential maintenance and dissemination of AMR determinants in farm environments	Monitor isolates from manure, bedding, wastewater, and mastitis cases
Adhesion-associated virulence factors	*yagZ*/*ecpA* and fimbrial genes	May promote mammary epithelial adhesion and early colonization, thereby increasing infection risk	Strengthen teat hygiene, milking sanitation, and environmental control
Iron acquisition systems	*fyuA*, *ybt*, *iuc*, iro-associated genes	May enhance survival in iron-limited host environments and contribute to persistent infection	Use as markers for high-risk strain screening and candidate vaccine antigen research
Capsule/biofilm-associated factors	*K-locus*, *wzi*/*wzc*, biofilm-associated genes	May enhance immune evasion, environmental persistence, and recurrent or chronic infection	Further assess capsule typing, biofilm phenotypes, and infection models
Open pangenome and abundant cloud genes	Cloud genes and mobile genetic element-associated genes	Indicate high genomic plasticity and ecological adaptability	Apply integrated control across cattle, bedding, water, manure, and milking environments

**Table 5:** Microorganisms or microbial groups associated with bovine-associated *K. pneumoniae* and their potential control relevance.

Microorganism or Microbial Group	Representative Taxa	Relationship with *K. pneumoniae*	Potential Control Relevance
Intestinal-associated microbiota	Ruminococcaceae, *Faecalibacterium*, Bacteroidales	Enriched in milk from cows with recurrent *Klebsiella* mastitis, suggesting fecal or intestinal microbial exposure	Indirect indicators of bedding, manure, and environmental hygiene status
Intestinal/environmental anaerobes	*Faecalibaculum*, *Anaerofustis*, *Muribaculum*, *Lachnoclostridium*	Associated with microbiota differences in recurrent *Klebsiella* mastitis cases	Highlight the need to reduce teat contact with manure, bedding, and contaminated environments
Environmental mastitis pathogens	*Escherichia coli*, *Streptococcus uberis*	Share similar environmental reservoirs and mastitis-control contexts with *K. pneumoniae*	Can be included in integrated environmental mastitis surveillance
Lactic acid bacteria/probiotic candidates	*Weissella cibaria*, *Lactobacillus* spp., *Lactococcus* spp.	Some strains may inhibit *K. pneumoniae* growth, adhesion, invasion, or inflammatory responses	Potential candidates for microbiota-oriented or antibiotic-sparing control strategies
Spore-forming and enterococcal probiotic candidates	*Bacillus* spp., *Enterococcus* spp.	May contribute to microbial competition or immune modulation, but strain-level safety is essential	Require safety assessment for AMR genes, virulence factors, and host compatibility before application
Mammary or teat commensal microbiota	Commensal bacteria from healthy mammary or teat-skin microbiota	May contribute to colonization resistance against environmental pathogens	Comparative metagenomics may help identify protective microbial markers

## Data Availability

The genome assemblies analyzed in this study were retrieved from the NCBI public database. The accession numbers, assembly information, genome file names, source metadata, MLST results, antimicrobial resistance-associated gene profiles, virulence-associated gene profiles, and plasmid replicon profiles of the included genomes are provided in Supplementary Tables S1–S4. No new genome sequences were generated in this study. To improve reproducibility, the metadata-screening workflow, including keyword-based bovine-source identification, metadata cross-checking, duplicate removal, and manual-curation criteria, has been described in detail in the Materials and Methods section. The custom Python scripts used for metadata parsing and curation are available from the corresponding author upon reasonable request.

## Supplementary Materials

The following supporting information can be downloaded at https://www.mdpi.com/article/10.3390/biology15141215/s1, Table S1 lists the complete accession numbers and associated metadata of the 1291 bovine-associated *K. pneumoniae* genomes included in this study, including assembly accession, assembly name, genome file name, NCBI genome FNA download link, strain name, collection year, country or region of origin, host, isolation source, MLST result, antimicrobial resistance-associated gene profile, virulence-associated gene profile, and plasmid replicon profile. Table S2 summarizes the pangenome composition, antimicrobial resistance-associated gene categories, virulence-associated gene categories, and plasmid replicon statistics. Table S3 provides the CARD-based antimicrobial resistance-associated gene profiles detected in bovine-associated *K. pneumoniae* genomes. Table S4 provides the VFDB-based virulence-associated gene profiles detected in bovine-associated *K. pneumoniae* genomes.
